# *Pseudocitrobacter cyperus, *a novel bacterial species recovered from *Cyperus alternifolius* in Egypt

**DOI:** 10.1186/s12866-024-03710-w

**Published:** 2025-01-14

**Authors:** Samira M. Hamed, Mai A. Amer

**Affiliations:** Microbiology and Immunology Department, October University for Modern Sciences and Arts (MSA), Giza, Egypt

**Keywords:** *Pseudocitrobacter*, Novel species, *Cyperus alternifolius*, Endophytes, Taxonogenomics, WGS

## Abstract

**Background:**

Strain Cyp38S^T^ was isolated as an endophyte from the plant *Cyperus alternifolius*, collected along the banks of the River Nile in 2019. Preliminary analysis tentatively identified Cyp38S^T^ as belonging to the genus *Pseudocitrobacter*.

**Methods:**

The preliminary identification of Cyp38S^T^ was performed using the VITEK^®^2 identification system, MALDI-TOF-MS, and 16S rRNA gene sequencing. To confirm its taxonomic classification, the draft genome of Cyp38S^T^ was generated using DNBseq, and the genome-based taxonomic evaluation was conducted by calculating the overall genome-relatedness indices (OGRIs) such as Average Nucleotide Identity (ANI), digital DNA-DNA hybridization (dDDH), and the tetra-nucleotide signatures (Tetra). Additionally, the biochemical features, antimicrobial susceptibility profiles, and fatty acid methyl ester content of Cyp38S^T^ were characterized.

**Results:**

VITEK^®^2 misidentified Cyp38S^T^ as *Citrobacter werkmanii*, MALDI-TOF-MS identified it as *Pseudocitrobacter faecalis.* While the 16S rRNA gene showed more than 99.0% similarity to other *Pseudocitrobacter* species, the calculated OGRIs were lower than the thresholds recommended for species assignment to all currently known *Pseudocitrobacter* species. Furthermore, the phylogenomic analysis revealed that Cyp38S^T^ forms a distinct species cluster within the genus *Pseudocitrobacter*. Cyp38S^T^ was predicted as a potential human pathogen and carried a unique ß-lactamase-coding gene.

**Conclusion:**

Here we present Cyp38S^T^ (= CCASU-2024-73^T^) as the type strain of a novel species within the genus *Pseudocitrobacter* to which we propose the name *Pseudocitrobacter cyperus* sp. nov. We provide a full description of the novel species and present its genome sequence and annotation. The discovery of this novel species highlights the potential of endophytic bacteria associated with unique plant hosts to harbor previously uncharacterized microbial diversity.

**Supplementary Information:**

The online version contains supplementary material available at 10.1186/s12866-024-03710-w.

## Introduction

*Pseudocitrobacter* is a fermentative, Gram-negative, facultatively anaerobic, rod-shaped bacteria lacking hemolytic activity and oxidase production.

The genus *Pseudocitrobacter* was established in 2014 and initially comprised two species, *P. faecalis* and *P. anthropi*, isolated from clinical specimens in Pakistan [1]. However, *P. anthropi* was later found to be a heterotypic synonym of *P. faecalis* [2]. Notably, some of the initial *Pseudocitrobacter* isolates were found to produce the carbapenemase enzyme New Delhi metallo-beta-lactamase (NDM-1) [1]. In 2020, a third species, *P. vendiensis*, was identified in a Danish patient repatriated from Spain, carrying a *Klebsiella pneumoniae* carbapenemase (KPC-2)-coding gene [2]. Subsequently, *P. corydidari*, the fourth species of *Pseudocitrobacter*, was isolated from the gut of a cockroach [3], highlighting the diverse ecological niches occupied by this bacterium. The latest species, *P. limerickensis*, was reported in 2024, isolated from a rectal swab of an inpatient in a hospital in Ireland. This strain also carried the *bla*_KPC−2_ gene [4]. The identification of *Pseudocitrobacter* species is challenging, as they are often initially misclassified as *Citrobacter* species.

Internationally, reports have highlighted the emergence of invasive *Pseudocitrobacter* infections. Several cases have been documented in Spain [5], Brazil [6], China [7], and Ireland [4] many of which were associated with the production of carbapenemase enzymes. These reports underscore the clinical significance of *Pseudocitrobacter* as a rapidly emerging pathogen that poses challenges in diagnosis and treatment.

Here, we report the characterization of an endophytic bacterial strain, designated Cyp38S^T^, isolated from the plant *Cyperus alternifolius*. Based on a comprehensive taxonogenomic analysis, the Cyp38S^T^ strain was found to be sufficiently distinct from other known *Pseudocitrobacter* species to warrant its classification as a novel species within the genus *Pseudocitrobacter*.

## Materials and methods

### Strain isolation and preliminary identification

Cyp38S^T^ was obtained as an endophyte from the leaves of *Cyperus alternifolius* plant collected along the banks of the River Nile in 2019, as part of a previous study [8]. The plant parts were collected from the Pharaonic Village (29.9973° N, 31.2148° E) in April 2019, with average temperatures ranging from 25 °C to 35 °C and humidity levels of 50–70%. Disease-free samples of mature and flowering *Cyperus alternifolius* were collected, stored in sealed plastic bags, and delivered to the laboratory on the same day for processing. The samples underwent surface sterilization as described before [8]. Briefly, plant parts were washed with tap and distilled water, dried with tissue paper, and then sterilized by sequential immersion in 70% ethanol (3 min), 1% sodium hypochlorite (12 min), and another ethanol rinse (30 s). This was followed by a three-step washing each for three minutes. After being dried in an airflow cabinet, the sterilized plant segments were cut into small pieces, placed on trypticase soy agar (TSA) plates, and incubated at 30 °C for 5 days. To ensure the sterilization was effective, the last rinse water was cultured on TSA and checked for contaminants. Distinct bacterial colonies were isolated and preserved in trypticase soy broth (TSB) with glycerol for further analysis as previously described [8].

Preliminary identification of the isolate involved Gram staining, oxidase test, and culturing on differential media such as MacConkey’s agar and Triple sugar iron agar (TSI). This was followed by Matrix-assisted laser desorption/ionization time-of-flight mass spectrometry (MALDI-TOF-MS) (Microflex spectrometer; Bruker Daltonics, Bremen, Germany). The mass spectra were processed using the default settings of the MALDI Biotyper Compass Explorer 4.1 software (Bruker Daltonics, Germany). Bacterial identification was based on matching protein mass spectral patterns against the Bruker database version 11, generating a logarithmic score between 0 and 3. According to the manufacturer’s recommendations, scores of 2 or higher indicate reliable species identification, scores between 1.7 and 1.999 indicate reliable genus identification, while scores below 1.7 are deemed unreliable.

Biochemical analysis were conducted using the VITEK^®^ 2 automated system (bioMérieux, Lyon, France). The isolated strain was cultured on TSA and incubated for 24 h at 37 °C. To prepare the samples, a uniform suspension with a microbial optical density ranging between 0.55 and 0.63 was achieved using a DensiCHEK™ VITEK^®^ 2 Caliper. Subsequently, the sample was analyzed using the VITEK^®^ 2 system, which involved performing 64 biochemical tests utilizing Gram-negative bacterium identification cards. Results were analysed using Vitek^®^ 2 Systems software version 9.03. Details of the biochemical tests conducted with the VITEK^®^2 system are outlined in Table 1.

### 16S rRNA-based identification and phylogeny

The genomic DNA from Cyp38S^T^ was isolated using the Quick-DNA Fungal/Bacterial Miniprep Kit from Zymo Research, following the manufacturer’s protocol. Subsequently, the 16S rRNA gene was amplified via PCR using the universal primers described before [9], and then sequenced bidirectionally using Sanger’s dideoxy DNA sequencing using ABI 3730xl DNA Analyzer (Applied Biosystems). The consensus sequence from the forward and reverse sequencing was assembled using Codon Code Aligner software (http://www.codoncode.com*).*

The partial gene sequence was used as input for pairwise sequence analysis on the 16S-based ID service provided by the EzBioCloud server [10] to identify the closely related type strains.

Subsequent alignment was performed with entries in the National Center for Biotechnology Information (NCBI) database (www.ncbi.nlm.nih.gov) utilizing the nucleotide Basic Local Alignment Search Tool (BLASTn). The search was conducted against the NCBI 16S ribosomal RNA database for Bacteria and Archaea type strains. This database contains curated 16S rRNA sequences from type strains of bacteria and archaea [11]. Only one representative type strain per species was included in our analysis. BLASTn search was also done against the NCBI nucleotide collection (nr/nt) database to identify the closest species whose genes were not deposited in the 16S ribosomal RNA database. Top hits with 100% coverage and at least 97.0% identity to the Cyp38S^T^ gene were selected for inclusion in the final phylogenetic tree. The genetic diversity was assessed using Molecular Evolutionary Genetics Analysis version 11.0 (MEGA 11.0) [12]. The phylogenetic tree was generated employing the Maximum Likelihood method and Tamura-Nei model [13], with 1000 bootstrap replicates [14]. The tree was visualized and edited using the interactive tree of life (iTOL) online tool v6.7 (https://itol.embl.de/itol.cgi*). Klebsiella pneumoniae* subsp. *pneumoniae* MGH 78,578 was employed as an outgroup.

### Whole genome sequencing and analysis

#### Library preparation and sequencing

Library construction and Whole Genome Sequencing (WGS) were performed at BGI Tech Solutions Hong Kong Co., Ltd. in China. The WGS process utilized DNBseq™ sequencing technology. Pre-assembly processing of the generated reads, including quality control and trimming, was conducted using SOAPnuke software developed by BGI [15].

#### Genome assembly, annotation, and functional characterization

The pre-processed reads underwent de novo assembly using Unicycler v. 0.4.8 [16]. The assembled contiguous sequences were then submitted to the NCBI Prokaryotic Genome Annotation Pipeline for gene annotation. The Clusters of Orthologous Group (COG) categories were assigned by searching against the Database of COGs of the NCBI [17] using eggNOG-mapper v2 [18].

#### Genome-based Taxonomy and Phylogenomics

To further investigate the taxonomic position of Cyp38S^T^, the draft genome sequence was analyzed using different genome-based taxonomy tools.

To determine the closest type strain genomes, the draft genomic sequence of Cyp38S^T^ was analysed using the Type Strain Genome Server (TYGS) by DSMZ (https://tygs.dsmz.de/) accessed on 20 October 2024 [19]. Using the MASH algorithm [20], a fast approximation of intergenomic relatedness, the draft genomic sequence was compared against all type strain genomes currently available in the TYGS database. The best matching type strains were used for constructing a phylogenomic tree. For the phylogenomic inference, all pairwise comparisons among the best-matching genomes were conducted using the Genome BLAST Distance Phylogeny approach (GBDP) [21], and accurate intergenomic distances were inferred under the algorithm ‘trimming’ and distance formula d5 [21]. The intergenomic distances obtained were utilized to construct a balanced minimum evolution tree using FASTME 2.1.6.1, with SPR post-processing [22]. Branch support was assessed through 100 pseudo-bootstrap replicates. The trees were rooted at the midpoint and visualized with PhyD3 [23].

The taxonomic placement of Cyp38S^T^ was also evaluated by calculating the overall genome-relatedness indices (OGRIs) for Cyp38S^T^ and other *Pseudocitrobacter* species, as recommended before [24, 25]. JSpeciesWS tool was used to determine the average nucleotide identity (ANI) with the alignment algorithms BLAST + (ANIb) and MUMmner (ANIm), as well as the correlation index of the Tetra-nucleotide signatures (Tetra) [26]. The digital DNA–DNA hybridization (dDDH) values were determined using the Genome-to-Genome Distance Calculator 3.0, accessible at *(*https://ggdc.dsmz.de/ggdc.php*)* [27].

#### Prediction of pathogenicity and detection of antimicrobial resistance genes and mobile genetic elements

The pathogenicity of Cyp38S^T^ was predicted using the PathogenFinder 1.1 tool [28]. Additionally, the antimicrobial resistance genes were predicted using the Resistance Gene Identifier (RGI) tool (v6.0.3) which uses the Comprehensive Antibiotic Resistance Database (CARD) (v3.3.0) as the reference database for known resistance determinants. Mobile genetic elements were screened using the MobileElementFinder tool (v1.0.3) [29].

#### Screening of the secondary metabolite biosynthetic gene clusters

To identify the secondary metabolite biosynthetic gene clusters (BGCs) present in the genome of Cyp38S^T^ compared to other *Pseudocitrobacter* species, we employed the antiSMASH bioinformatic tool (version 7.0) using a relaxed detection strictness and default parameters [30]. All genome sequences were provided as input to antiSMASH, which scanned the genomes for the presence of signature biosynthetic genes and identified the surrounding gene clusters potentially involved in secondary metabolite production.

#### Screening of plant interaction genes

Plant interaction genes were screened using the PIFAR-Pred tool available on the PLant-associated BActeria web resource (PLaBAse) (https://plabase.cs.uni-tuebingen.de/pb/form.php?var=PIFAR-Pred), with default settings applied [31]. The tool predicts plant-associated bacteria by annotating around 123 proteins linked to plant interactions using the blastp + hmmer method. The tool is based on a machine-learning model developed by Martínez-García, et al. [32]. Our analysis focused on Cyp38S^T^ and type strains from other *Pseudocitrobacter* species. Additionally, we included *P. faecalis* RIT415 (GenBank accession: QBJC00000000), an endophytic bacterium isolated from sugarcane [33].

### Pan-genome analysis of *pseudocitrobacter* genomes

The pan-genome analysis was performed using all available genomes belonging to the genus *Pseudocitrobacter* in the NCBI genome database at the time of the study (30.05.2024). This included a total of 12 genomes, with the genome sequence from the Cyp38S^T^ strain obtained as part of the current work.

The pan-genome of the genus *Pseudocitrobacter* was constructed using Roary v3.13.0 [34]. Prior to the pan-genome analysis, the draft genomes were reannotated using Prokka v1.14.5 [35] to generate the necessary GFF files. Roary was then used to create a matrix of gene presence and absence across the genomes, which was visualized using Roary plots. The genes unique to Cyp38S^T^ were extracted from the pan-genome using the query_pan_genome script. Finally, Kyoto Encyclopedia of Genes and Genomes (KEGG) Orthology (KO) groups were assigned to the unique genes of Cyp38S^T^ using GhostKOALA [36]. The same tool was used for exploring the pathway modules in Cyp38S^T^.

### Accession numbers

The partial sequence of the 16S rRNA gene was submitted to the NCBI database with the accession number PQ461854.2. The Whole Genome Shotgun project has been also deposited in the NCBI database under the BioProject number PRJNA1060458 and the genome assession number JAYMYY000000000.

### Phenotypic characterization

The growth of Cyp38S^T^ was examined at temperatures ranging from 4° to 45 °C on TSA under anaerobic and aerobic conditions. Cyp38S^T^ was also cultured on other commonly used culture media such as blood agar, nutrient agar, Mueller-Hinton agar, and MacConkey’s agar. The strain’s ability to sporulate was tested through thermal shock at 80 °C for 30 min. Additionally, the strain’s tolerance to various salinity levels (0–10% NaCl) and pH conditions (4–10) was investigated [37]. Enzymatic activities such as catalase and oxidase were analyzed as described before [38]. The strains’ biochemical characteristics were analyzed using VITEK^®^2 which was also used for testing the susceptibility of Cyp38S^T^ to a panel of 14 antimicrobial agents including piperacillin/tazobactam, cefazolin, cefoxitin, ceftazidime, ceftriaxone, cefepime, meropenem, amikacin, gentamicin, tobramycin, ciprofloxacin, levofloxacin, nitrofurantoin, and trimethoprim/sulfamethoxazole. All tests were done according to the manufacturer’s instructions. The antimicrobial susceptibility test was interpreted according to the Clinical and Laboratory Standards Institute (CLSI) guideline [39].

### Gas Chromatography-Mass Spectrometry (GC-MS) fatty acid methyl ester (FAME) analysis

Fatty acid methyl ester (FAME) analysis was performed using Gas Chromatography/Mass Spectrometry (GC/MS). The preparation of FAMEs followed the protocol described by Sasser and colleagues [40]. The GC-MS analysis was done as described before [8].

## Results

### Preliminary identification of Cyp38S^T^

The results of conventional microbiological identification of Cyp38S^T^ were consistent with *Enterobacteriaceae*. Gram staining revealed short, scattered, Gram-negative cells. Cyp38S^T^ tested oxidase-negative. On MacConkey agar, it produced lactose-fermenting colonies, and growth on TSI agar showed an alkaline slant with an acidic butt.

**Fig. 1:**
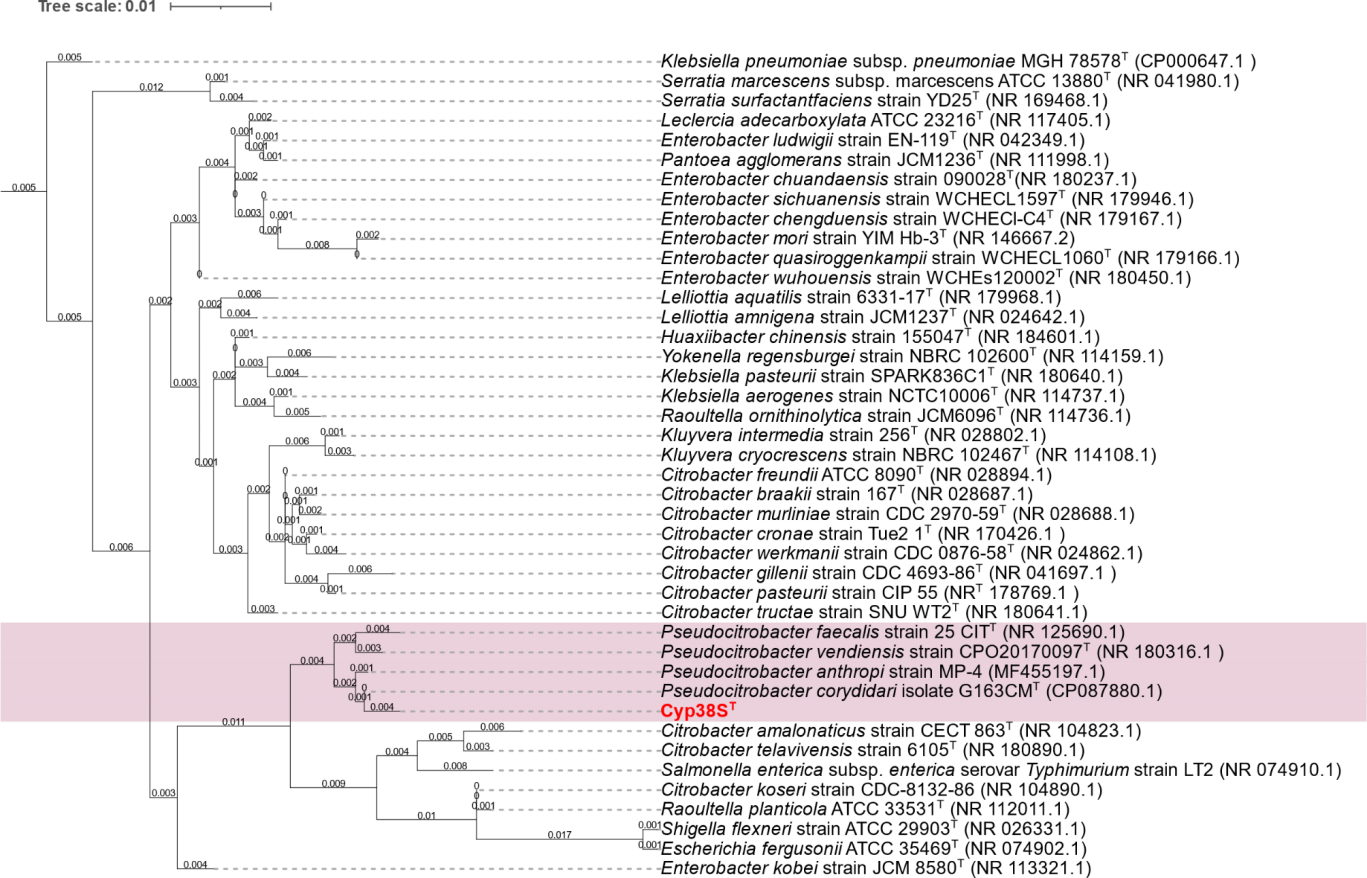
Phylogenetic tree based on 16S rRNA gene sequences showing the taxonomic position of Cyp38S^T^ among the closely related bacterial species. The novel species type strain proposed in this study, Cyp38S^T^, was highlighted in bold red within the tree. The superscript “T” indicates the type strains of each species. The tree was constructed from the alignment of 1407 bp sequences. GenBank accession numbers are given in parentheses

Cyp38S^T^ was identified by VITEK^®^2 with a high confidence level (93%) as *Citrobacter werkmanii*. However, this identification conflicted with the result from MALDI-TOF-MS, which identified it as *P. faecalis*. Consequently, the isolate was further identified using 16S rRNA gene sequencing. Using the EzBioCloud 16S-based identification service, Cyp38S^T^ exhibited the highest similarity to *P. corydidari* strain G163CM^T^, with a similarity of 99.15%. BLAST analysis of the 16S rRNA gene of Cyp38S^T^ against the 16S ribosomal RNA database for Bacteria and Archaea type strains, as well as the nucleotide collection (nr/nt) database, revealed high similarity to the type strains of other *Pseudocitrobacter* species, with identity percentages ranging from 98.93 to 99.57%. The phylogenetic tree, constructed using 16S rRNA sequences from the closest matching type strains (Identity ≥ 97.0%), confirmed that Cyp38S^T^ is a unique member of the genus *Pseudocitrobacter*, as shown in Fig. 1.

### Genome features of Cyp38S^T^

**Fig. 2:**
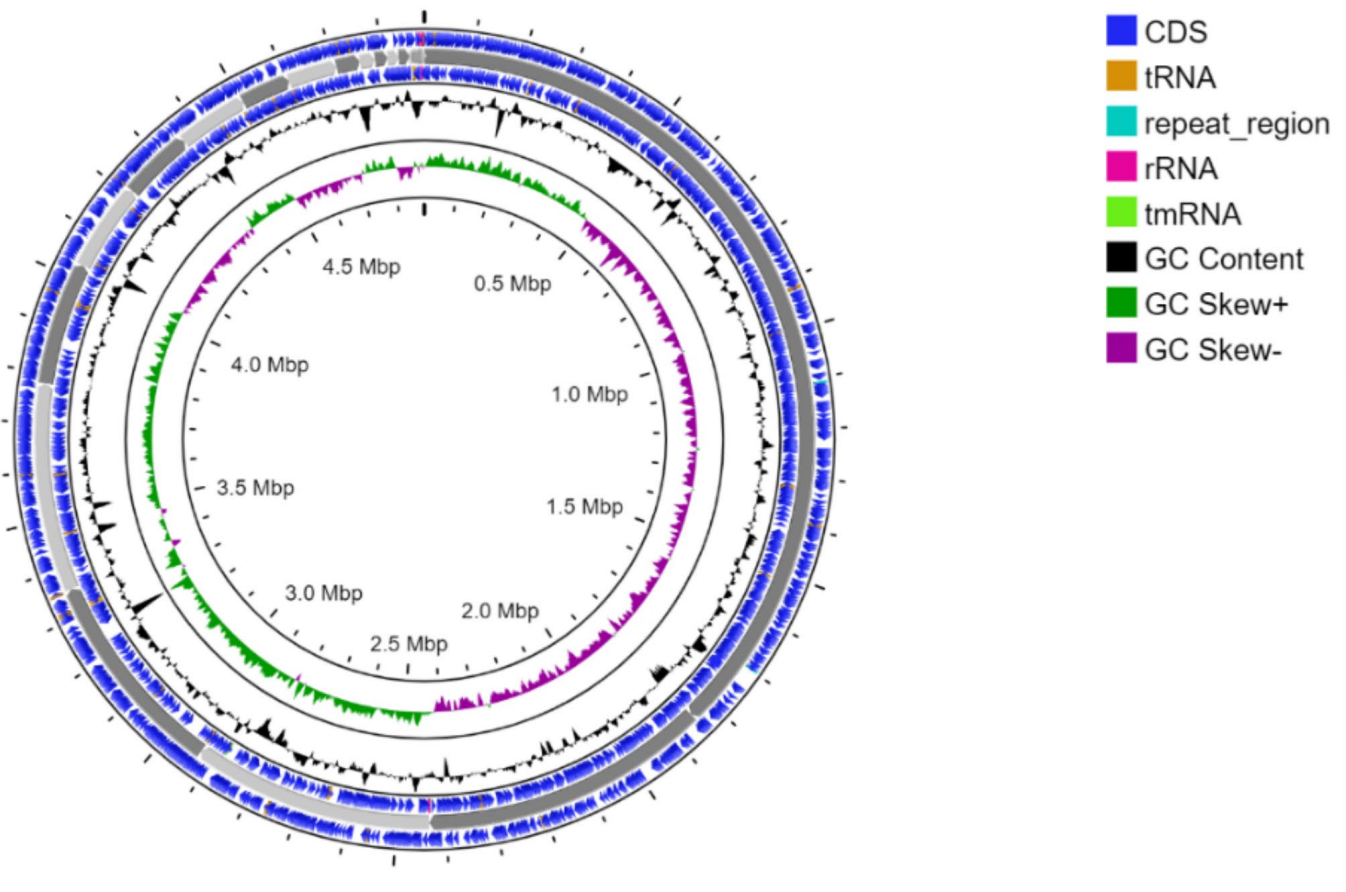
Circular map of Cyp38S^T^ genome created by Proksee

The de novo assembled draft genome of Cyp38S^T^ comprised 33 scaffolds with a total length of 4,887,821 bp, N_50_ of 493,251 bp, and L_50_ of 3. The GC content is 53.4%. Genome analysis showed good quality with 99.8% completeness and 0.2% contamination. The genome was found to encode approximately 4,477 protein-coding sequences, as well as 91 RNA genes, including 6 rRNA, 77 tRNA, and 8 non-coding RNA genes. No plasmids were identified in the draft genome, but a CRISPR array was detected, suggesting the presence of a functional CRISPR-Cas system in this bacterial strain. A circular map of the genome is shown in Fig. 2.

### Phylogenomic features and genome-based taxonomy of Cyp38S^T^

**Fig. 3:**
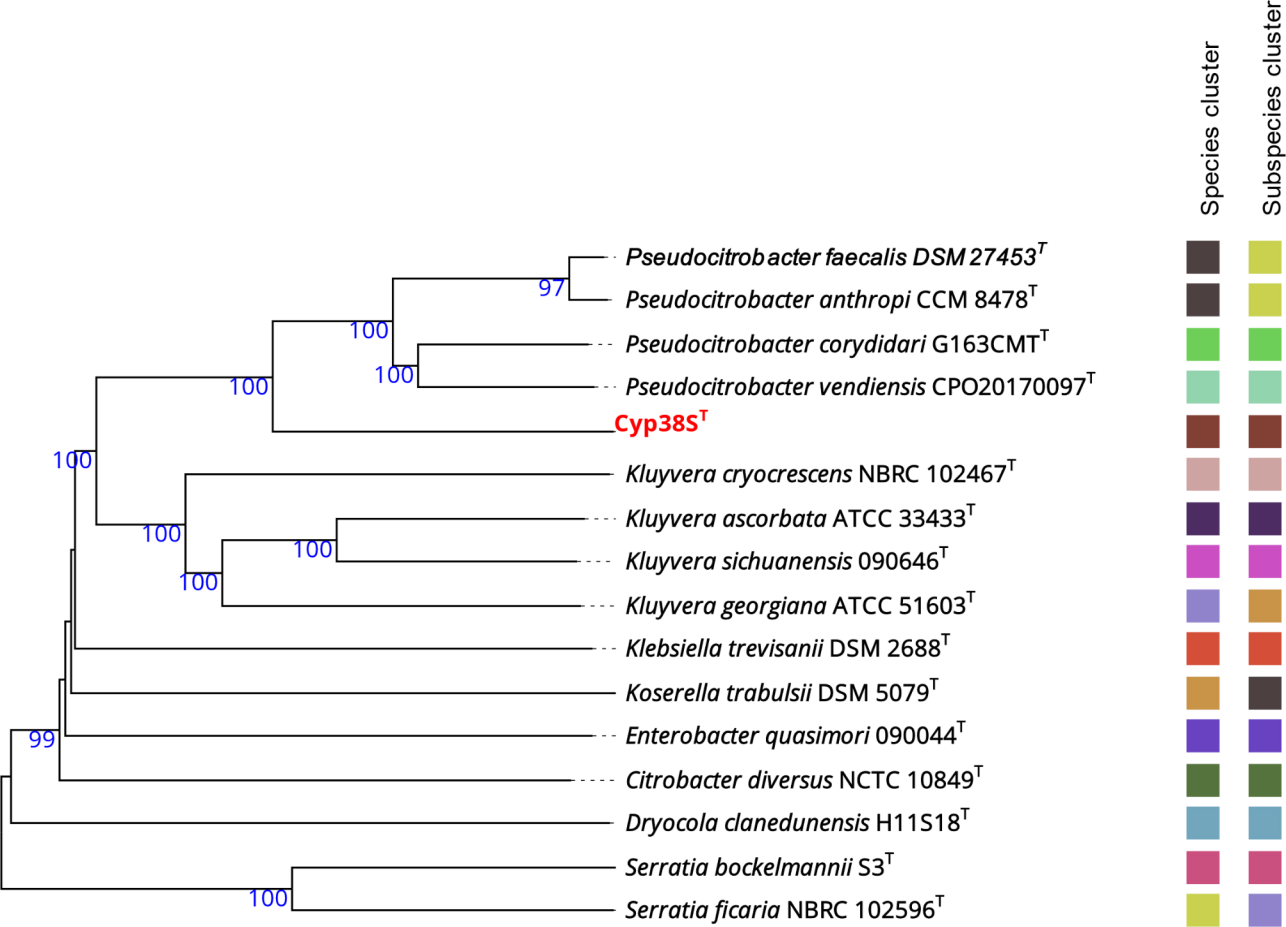
Phylogenomic tree constructed from GBDP distances calculated from the genomes of the TYGS type strains most closely related to Cyp38S^T^. Species and subspecies clusters are defined by 70% and 79% dDDH thresholds, respectively. The numbers above the branches represent GBDP pseudo-bootstrap support values from 100 replicates. The tree is midpoint-rooted. The proposed novel species type strain, Cyp38S^T^, is highlighted in bold red within the tree. The superscript “T” indicates the type strains of each species

To further elucidate the phylogenomic placement of the Cyp38S^T^ strain, the draft genome was compared to the genomes of all type strains available in the TYGS database. The generated phylogenomic tree, depicted in Fig. 3, showed that Cyp38S^T^ forms a distinct species cluster within the genus *Pseudocitrobacter* (based on 70% dDDH threshold), indicating that it represents a type strain for a novel species not closely related to the currently described species of *Pseudocitrobacter*.

To further confirm the proposed taxonomic placement of strain Cyp38S^T^ as the type strain of a novel species within the genus *Pseudocitrobacter*, the OGRIs were calculated for Cyp38S^T^ and type strains of all currently known *Pseudocitrobacter* species. All ANI and dDDH values were consistently below the respective species delineation thresholds of 95% and 70% recommended by Chun, et al. [41]. Additionally, Tetra values did not exceed the cut-off of 0.999 set by the JSpeciesWS server, as shown in Table 1.

**Table 1:** Overall genome-relatedness indices (OGRIs) calculated for Cyp38S^T^ compared to the type strains of all currently known *Pseudocitrobacter* species

*Pseudocitrobacter* species type strains	OGRIs of Cyp38S^T^ compared to other *Pseudocitrobacter* species			
	ANIb	ANIm	dDDH	Tetra
*P. faecalis* DSM 27,453^T^	86.4	88.2	33.0	0.99
*P. anthropi* CCM 8478^T^	86.6	88.2	33.0	0.99
*P. corydidari* G163CM^T^	87.0	88.7	34.7	0.98
*P. vendiensis* CPO20170097^T^	86.6	88.4	33.5	0.99

ANIb, the average nucleotide identity with the alignment algorithm BLAST +; ANIm, the average nucleotide identity with the alignment algorithm MUMmer; Tetra, the correlation index of the Tetra-nucleotide signatures. The superscript “T” indicates the type strains of each species

### COG assignment of Cyp38S^T^ genes compared to other *Pseudocitrobacter* species

**Fig. 4:**
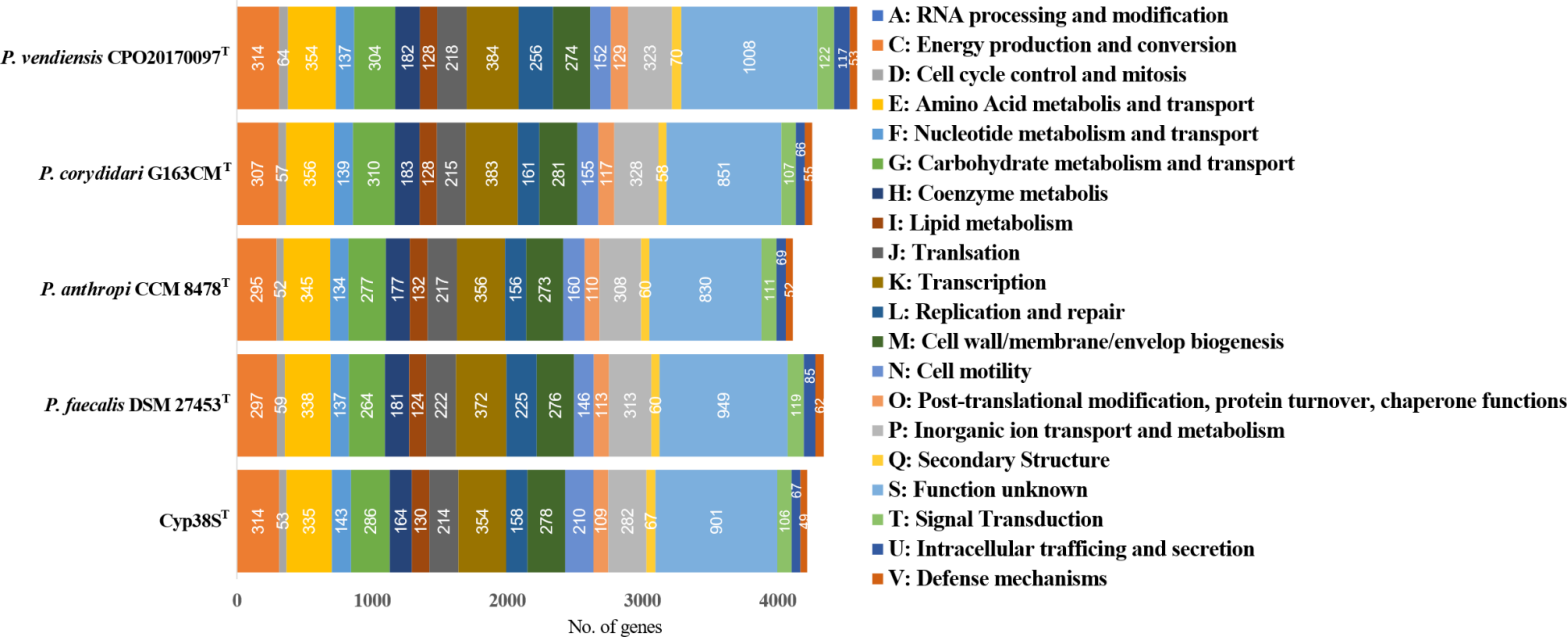
Number of genes associated with COG functional categories in Cyp38S^T^ compared to the type strains of other *Pseudocitrobacter* strains. The superscript “T” indicates the type strains of each species

To further understand the genomic features of Cyp38S^T^, the number of genes assigned to different COGs was determined and compared to the type strains of other *Pseudocitrobacter* species (Fig. 4). Interestingly, Cyp38S^T^ was found to have the highest number of genes related to cell motility among the analyzed strains. In contrast, Cyp38S^T^ carried the second-lowest number of genes associated with defense mechanisms within the genus *Pseudocitrobacter*.

### Pathogenicity, antimicrobial resistance genes, and mobile genetic elements carried by Cyp38S^T^

The PathogenFinder tool assessed Cyp38S^T^ as having an 80.3% probability of being a human pathogen, with its proteome matching 64 pathogenic families. Using an identity cutoff of 85%, the RGI tool identified antimicrobial resistance genes in Cyp38S^T^. Notably, Cyp38S^T^ carries a gene coding a novel CMY2/MIR/ACT/EC family class C beta-lactamase. Moreover, Cyp38S^T^ harbors genes encoding the antibiotic efflux pumps MsbA, AcrAB-TolC, MdtABC-TolC, AcrD, MdtG, EmrD, EmrAB-TolC, and MdtK. Upon uploading the draft genome into the MobileElementFinder tool, it was determined that Cyp38S^T^ does not carry any mobile genetic elements.

### Secondary metabolite BGCs and plant interaction genes

To explore the genetic potential for secondary metabolism in the novel species identified here compared to other *Pseudocitrobacter* species, we utilized antiSMASH to identify secondary metabolite BGCs across various strains. The analysis revealed that all investigated strains contained the same four BGCs, which encoded a non-ribosomal peptide synthetase (NRPS), a thiopeptide, a ribosomally synthesized and post-translationally modified peptide product (RiPP), and an aryl polyene.

**Fig. 5:**
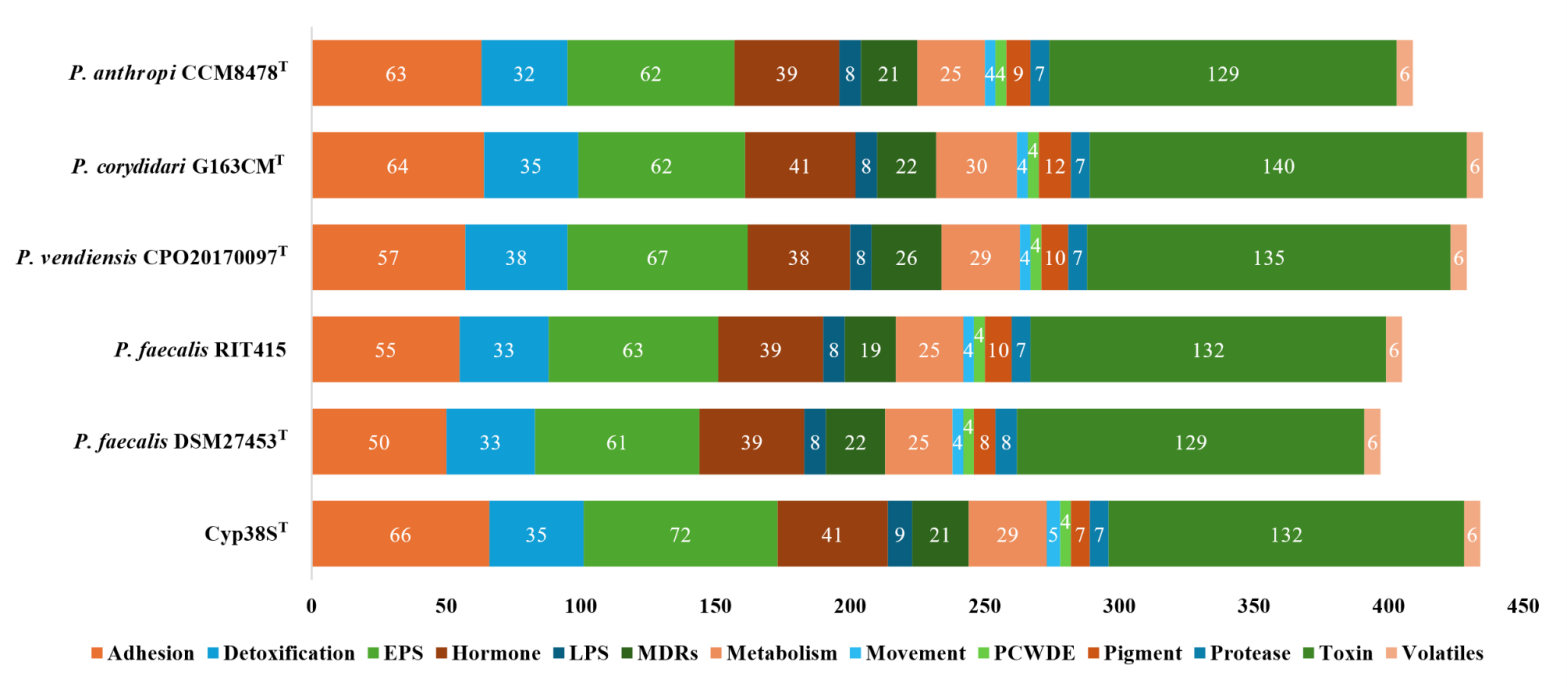
Plant interaction genes identified in Cyp38S^T^ using the PIFAR-Pred tool, in comparison to other *Pseudocitrobacter* strains. The superscript “T” indicates the type strains of each species. EPS, exopolysaccharide; LPS, lipopolysaccharide; MDRs, multidrug resistance genes; PCWDE, Plant cell wall degrading enzymes

The analysis of plant-associated genes using the PIFAR-Pred tool revealed that Cyp38S^T^ shares numerous key genes with other *Pseudocitrobacter* species, all of which play essential roles in plant interactions. A total of 434 plant interaction genes were identified in Cyp38S^T^. The predicted protein products of these genes are involved in critical functions, including adhesion, detoxification, exopolysaccharide (EPS) production, hormone synthesis (such as indole acetic acid and cytokinin), motility, pigment synthesis (such as zeaxanthin diglucoside), plant cell wall degrading enzymes (PCWDEs), toxin production (e.g., syringomycin, syringopeptin, and riboflavin), and volatile compound synthesis. Figure 5 illustrates the classes of plant interaction genes identified in Cyp38S^T^ compared to the type strains of other *Pseudocitrobacter* species.

The gene count comparison across *Pseudocitrobacter* strains highlights significant differences in their potential interactions with plant hosts or environments. Cyp38S^T^ exhibits the highest number of genes related to adhesion (66), EPS production (17), and movement (5), suggesting a stronger capability for plant root colonization. *P. corydidari* G163CM^T^ and *P. vendiensis* CPO20170097^T^ have a higher count of toxin-related genes (32 and 33, respectively), indicating high pathogenic potential. Additionally, *P. faecalis* DSM27453^T^ is unique in possessing two genes linked to volatile compound production (Supplementary Table 1). Although these strains share many similarities, these variations may impact their specific interactions with their environments.

### Pan-genome features of *Pseudocitrobacter* species

**Fig. 6:**
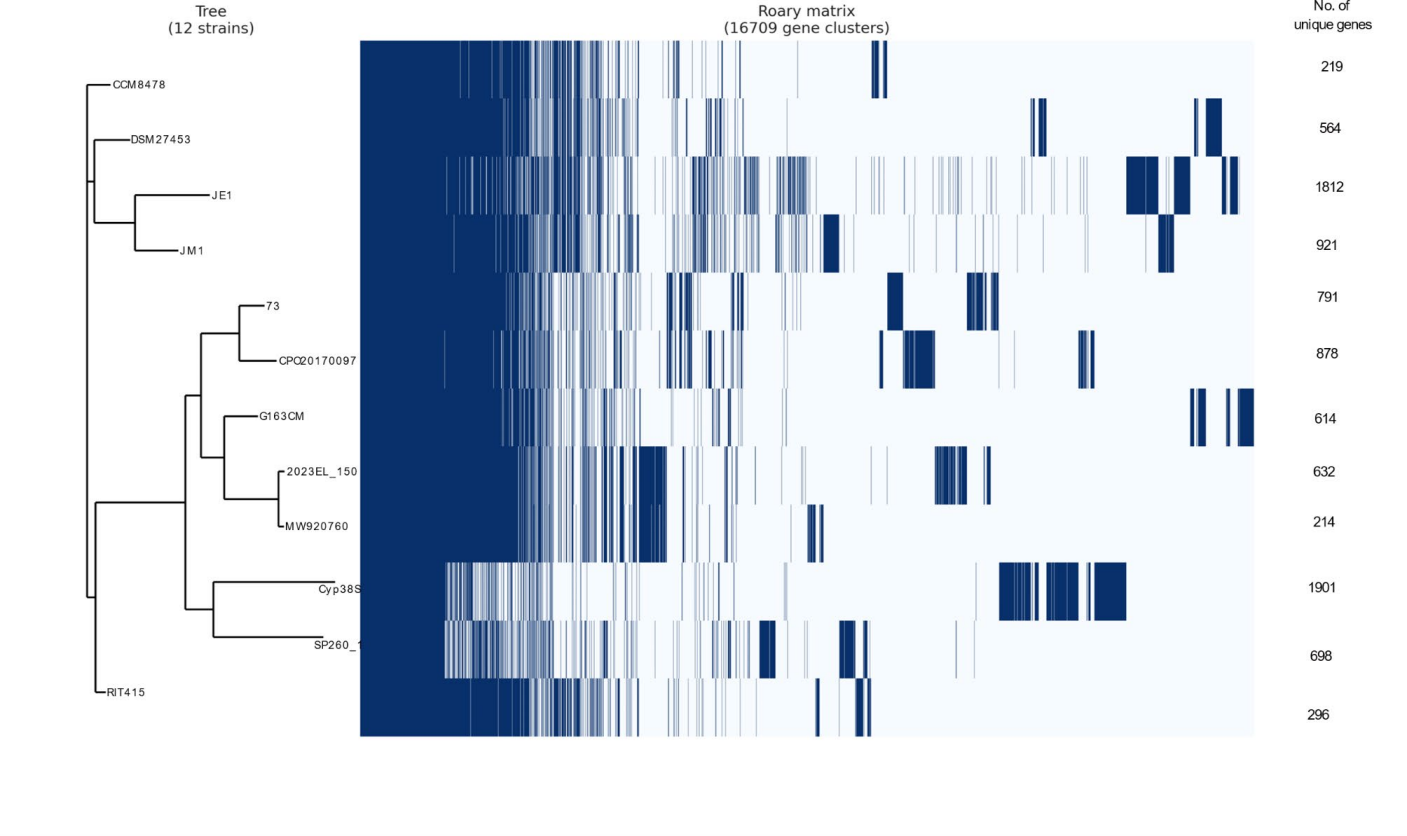
Gene presence/absence matrix from the pangenome analysis of 12 *Pseudocitrobacter* strains including Cyp38S^T^

The pan-genome of the studied *Pseudocitrobacter* strains comprised 16,709 genes including 1,575 core genes and 15,134 accessory genes. Among all analysed strains, Cyp38S^T^ carried the highest number of unique genes (1,901 genes), as shown in Fig. 6.

GhostKOALA analysis identified 101 KEGG pathway modules in Cyp38S^T^. The signature KEGG modules characterizing the phenotypic features of Cyp38S^T^ revealed a metabolic capacity for nitrate and sulfur-sulfur assimilation. In addition to complete pathways for carbohydrate, energy, lipid, nucleotide, amino acid, and glycan metabolism, Cyp38S^T^ was rich in pathways for the production of cofactors, vitamins, and terpenoids. Additionally, Cyp38S^T^ carried complete pathway modules for the degradation of aromatic compounds such as benzoate, catechol, phenylacetate, and trans-cinnamate. All complete pathway modules identified in Cyp38S^T^ are listed in Supplementary Table 2.

Out of the 1901 predicted proteins identified by Roary as unique to Cyp38S^T^ compared to other *Pseudocitrobacter* species, only 873 (45.9%) were assigned to KO groups (Supplementary Table 3). Notably, 60 out of 64 predicted bacterial motility proteins in Cyp38S^T^ were unique to this novel species (Supplementary Table 4). Among the 101 complete pathway modules predicted in Cyp38S^T^, two were unique compared to other species: one involved in nitrogen metabolism (assimilatory nitrate reduction) and the other in arginine metabolism (arginine succinyltransferase pathway) (Supplementary Table 2).

The taxonomic assignment of the unique predicted proteins showed that 1,886 proteins were linked to bacterial species, 12 to viruses, and only one to Archaea. The majority of unique proteins (1,811) were classified within *Enterobacteria*, predominantly associated with the genera *Pseudocitrobacter* (567 proteins), *Enterobacter* (128), *Escherichia* (66), and *Citrobacter* (65). Interestingly, 565 predicted proteins remained unassigned to any specific genus within *Enterobacteria* (Supplementary Table 5). The KO groups of all unique proteins not assigned to any known genus are listed in Supplementary Table 6.

### Phenotypic features of Cyp38S^T^

The colonies of Cyp38S^T^ were observed to be small, glistening, and creamy white in color. Microscopic examination revealed that the cells were scattered, rod-shaped, and Gram-negative, with no spore formation. It exhibited positive growth under both aerobic and anaerobic conditions within 24 h and was able to grow across all tested temperatures, pH levels, and salinity concentrations. Cyp38S^T^ showed non-hemolytic growth on blood agar and grew well on other culture media including nutrient agar, Mueller Hinton’s agar, and MacConkey’s agar. The novel species was catalase-positive but showed negative oxidase activity. The full biochemical profile of Cyp38S^T^ characterized by VITEK^®^2 is shown in Table 2. Testing the antimicrobial susceptibility of Cyp38ST revealed that the strain was sensitive to all of the tested antibiotics, except cefazolin (MIC = ≥ 8 mg/L) and cefoxitin ((MIC = ≥ 32 mg/L).

**Table 2:** Detailed biochemical characteristics of Cyp38S^T^

Test	Result	Test	Result
Ala-phe-pro-arylamidase	**-**	Saccharose/Sucrose	**-**
Adonitol	**-**	D-tagatose	**-**
L-pyrrolydonyl-arylamidase	**+**	D-trehalose	**+**
L-arabitol	**-**	Citrate (sodium)	**-**
D-cellobiose	**+**	Malonate	**+**
ß-galactosidase	**+**	5-keto-D-gluconate	**+**
H_2_S production	**-**	L-lactate alkalinisation	**+**
ß-N-acetyl-glucoseaminidase	**-**	alpha-glucosidase	**-**
Glutamyl arylamidase pNA	**-**	Succinate alkalinisation	**+**
D-glucose	**+**	ß-N-acetyl-galatosaminidase	**-**
Gamma- glutamyl- transferase	**-**	alpha-galactosidase	**-**
Fermentation/ glucose	**+**	Phosphatase	**+**
ß-glucosidase	**-**	Glycine arylamidase	**-**
D-maltose	**+**	Ornithine decarboxylase	**-**
D-mannitol	**+**	Lysine decarboxylase	**-**
D-mannose	**+**	Decarboxylase base	**-**
ß-xylosidase	**+**	L-histidine assimilation	**-**
ß-alanine arylamidase pNA	**-**	Coumarate	**-**
L-proline arylamidase	**-**	ß-glucoronidase	**-**
Lipase	**-**	O/129 resistance (comp.*vibrio.*)	**+**
Palantinose	**-**	Glu-Gly-Arg- arylamidase	**-**
Tyrosine arylamidase	**+**	L-malate assimilation	**-**
Urease	**-**	Ellman	**+**
D- sorbitol	**+**	L-lactate assimilation	**-**

### Cellular fatty acid profile of Cyp38S^T^

Based on FAME GC-MS analysis, the most abundant fatty acids in Cyp38S^T^ were found to be C_16:0_, C_17:1_ n-7 cis, and C_18:1_ n-9. Details about the fatty acid composition of Cyp38S^T^ compared to other measured in *Pseudocitrobacter* species are shown in Table 3.

**Table 3:** Cellular fatty acid composition (percentages) of Cyp38S^T^ analyzed by GC-MS compared to other *Pseudocitrobacter* species

Lipid number	Cyp38S^T^	*P*. *corydidari*	*P*. *vendiensis*	*P*. *faecalis*	*P*. *limerickensis*
C_12:0_	Not detected	2.6	3.6	3.7	3.7
C_14:0_	6.8	6.5	6.6	7.2	9.3
C_14:1_	5.7	Not detected	Not detected	Not detected	Not detected
C_15:0_	2.3	1.0	1.4	1.1	Not detected
C_15:1_ n-5 cis	0.7	Not detected	Not detected	Not detected	Not detected
iso-C_15:0_	Not detected	Trace	Not detected	Not detected	Not detected
C_16:0_	37.1	31.5	30.9	28.4	36.3
C_16:1_	7.9	Not detected	Not detected	Not detected	Not detected
C_17:0_ cyclo	0.9	15.3	18.6	13.2	20.8
C_17:1_ n-7 cis	21.8	Not detected	Not detected	Not detected	Not detected
C_18:0_	1.2	Not detected	Not detected	Not detected	Not detected
C_18:1_ n-9	10.3	22.6	20.8	22.9	3.7
C_18:2_ n-6,9 all-cis	0.4	Not detected	Not detected	Not detected	Not detected
C_18:3_ n-6,9,12 all-cis	4.9	Not detected	Not detected	Not detected	Not detected
C_19:0_ cyclo ω8c	Not detected	2.5	2.6	5.5	7.8
Summed Feature 2	6.8	9.4	8	8.1	7.5
Summed Feature 3	7.9	8.6	6.6	9.1	3.9

## Discussion

The present study describes the isolation and comprehensive characterization of a novel bacterial strain, Cyp38S^T^, obtained as an endophyte from the plant *Cyperus alternifolius*. Through a polyphasic taxonomic approach, including phenotypic, genotypic, and phylogenetic analyses, we demonstrate that Cyp38S^T^ represents a distinct species within the genus *Pseudocitrobacter*.

As previously discussed [1–3], the discrepancy in the identification results from VITEK^®^2 and MALDI-TOF-MS, highlighted the challenges in accurately classifying Cyp38S^T^ and underscored the limitations of relying solely on phenotypic and biochemical tests for the identification of *Pseudocitrobacter* species, which can often be misclassified as members of the closely related *Citrobacter* genus.

16S rRNA-based identification revealed that Cyp38S^T^ exhibited sequence identities exceeding 99.0% with other species within the genus *Pseudocitrobacter*. This level of similarity surpasses the recently recommended cutoff value for species differentiation of 98.65% [42]. Notably, similar high 16S rRNA identity values have been documented among type strains of various *Pseudocitrobacter* species. For instance, a BLAST analysis conducted by Kämpfer, et al. [2] demonstrated that the type strain of *P. vendiensis* (CPO20170097^T^) showed over 99.2% identity with the type strains of *P. faecalis* and *P. anthropi*. Additionally, the 16S rRNA gene sequence of G163CM^T^, the type strain of *P. corydidari*, displayed a similarity of 99.22% to both *P. faecalis* CCM 8479^T^ and *P. vendiensis* CPO20170097^T^. These findings underscore the challenges in using 16S rRNA gene sequence similarity as a definitive metric for species delineation within this genus.

The phylogenomic tree created by TYGS for Cyp38S^T^ and the type strains of all currently known species showed that it formed a distinct species cluster among the type strains of the genus *Pseudocitrobacter*. Interestingly, the phylogenomic tree created by TYGS has placed the type strains of *P. faecalis* and *P. anthropi* within the same species and subspecies clusters, confirming previous recommendations for revising the taxonomy of *P. anthropi*, suggesting that it should be classified under the species *P. faecalis*. This further emphasizes the necessity for genomic approaches in accurately resolving taxonomic relationships among closely related bacterial species [2]. The taxonomic placement of Cyp38S^T^ was further confirmed by calculating various OGRIs between Cyp38S^T^ and the type strains of all currently recognized *Pseudocitrobacter* species. ANI and dDDH values consistently fell below the species delineation thresholds of 95% and 70% recommended by Chun, et al. [41]. Although relatively high Tetra values (ranging from 0.98 to 0.99) were observed for Cyp38S^T^ and other type strains, they did not exceed the species delineation cut-off of 0.999 set by the JSpeciesWS server. The observation of high Tetra values alongside lower ANI values has been attributed in previous studies to evolutionary or environmental pressures that restrict changes in genome signatures, even in the presence of genetic drift [43].

Notably, the genome analysis of Cyp38S^T^ revealed the presence of a unique β-lactamase-encoding gene, as well as efflux pump genes. This finding, coupled with the prediction of Cyp38S^T^ as a potential human pathogen, underscores the clinical relevance of this novel species and the need for improved surveillance and identification methods to manage emerging *Pseudocitrobacter* infections.

The discovery of this novel endophytic *Pseudocitrobacter* species highlights the potential of underexplored ecological niches, such as plant-associated microbiomes, to harbor previously uncharacterized microbial diversity. Endophytic *Pseudocitrobacter* RIT415 has been previously isolated from sugarcane on the island of Jamaica [31]. Additionally, *Pseudocitrobacter* was isolated from the rhizosphere of soybean in Pakistan [32]. As *Pseudocitrobacter* has previously been recovered from patients with invasive infections, occasionally carrying carbapenemase-coding genes, the finding of this species as an endophyte raises questions about the possibility of transfer of this bacterial species to humans through plants. It’s interesting to note that *Kluyvera* species, which are frequently found in endophytic and rhizosphere environments, have been suggested as the initial source of *bla*_CTX−M_-type genes [44]. Thus, endophytic bacteria colonizing the plant tissues may be a covert method for humans and other animals to come into exposure to virulent and antibiotic-resistant bacteria [45]. Plants can be colonized by human pathogenic microorganisms either through natural openings such as stomata, lenticels, hydathodes, and sites of lateral root formation, as well as through areas of physical and chemical damage [46]. The range of pathogens that can colonize plants encompasses *Escherichia coli* O157-H7, *Salmonella* sp., *Shigella* sp., *Listeria monocytogenes*, *Clostridium botulinum*, *Campylobacter* sp., *Bacillus cereus*, *Aeromonas hydrophila*, *Vibrio cholerae*, and *Yersinia enterocolitica* [47].

On the other side, the genome analysis of Cyp38S^T^ revealed a diverse array of secondary metabolites produced by this novel species. Furthermore, Analysis of the plant interaction genes carried by Cyp38S^T^ showed that The genomic analysis of Cyp38S^T^, when compared to other *Pseudocitrobacter* strains, revealed notable variations that may significantly influence their interaction with plant hosts. Cyp38S^T^ exhibits the highest adhesion, EPS production, and motility gene counts, which likely enhances its ability to colonize plant roots and other plant tissues [48–50]. In contrast, *P. cordyradi* and *P. vendiensis* display a higher number of toxin-related genes, indicating a potential for stronger pathogenicity or defense mechanisms in competitive environments. All strains could produce volatile compounds, a feature often associated with signaling and interaction with plant hosts or other microbes [51]. The occurrence of plant-interaction genes in both clinical isolates and environmental strains of *Pseudocitrobacter* species can be attributed to factors like horizontal gene transfer, which allows for the acquisition of beneficial traits from diverse environments. Our analysis highlights that, although core functions for plant association are conserved across all *Pseudocitrobacter* species, each strain exhibits unique adaptations that may shape its ecological role, from beneficial symbiosis to potential pathogenicity [52].

Cyp38S^T^ genome contained complete pathway modules for the degradation of aromatic compounds, underscoring the significant biotechnological potential of this species for both the biosynthesis of secondary metabolites and applications in biodegradation. In a recent study by Husna, et al. [53], a rhizobacterial isolate of *P. anthropi* demonstrated significant potential for bioremediation and plant growth promotion under heavy metal stress. These findings warrant the need for exploring the biotechnological potential of the novel strain Cyp38S^T^, which may offer additional benefits in the context of environmental remediation and plant health.

The biochemical characterization of strain Cyp38S^T^, in comparison to other *Pseudocitrobacter* species [1, 2], highlights both conserved traits and distinct differences that could aid in species identification. All species consistently tested negative for adonitol, H₂S production, ß-N-acetyl-glucosaminidase, urease, and lipase. Conversely, positive results were noted for D-cellobiose, D-trehalose, D-mannitol, D-mannose, and glucose fermentation. A significant distinction is that Cyp38S^T^ exhibited negative citrate activity, whereas all other species tested positive. Additionally, Cyp38S^T^ was positive for D-sorbitol fermentation, while the other strains showed negative results. The fatty acid profile of Cyp38S^T^ shows notable distinctions from other species in the genus, such as *P. faecalis*, *P. vendiensis*, and *P. corydidari*. Cyp38S^T^ has a higher proportion of C_16:0_ (37.2%) and C_17:1_ n-7 cis (21.8%), while exhibiting lower levels of C_18:1_ ω7c (10.3%) and an absence of C_19:0_ cyclo ω8c, which are more prominent in other species. These biochemical differences may aid in the identification and differentiation of the novel species *Pseudocitrobacter cyperus* from closely related species.

## Conclusion

Based on the data generated in this study, we propose Cyp38S^T^ as the type strain of a novel species within the genus *Pseudocitrobacter* to which we propose the name *Pseudocitrobacter cyperus* sp. nov. The discovery of this novel species highlights the importance of exploring underexplored microbial niches and the application of advanced genomic techniques for the identification and classification of clinically relevant bacteria. Furthermore, the genomic data generated in the current study warrant the need to investigate the biotechnological potential of Cyp38S^T^.

### Description of *pseudocitrobacter cyperus sp*. nov

*Pseudocitrobacter cyperus* (cy.per’us. N.L. masc. adj. *cyperus*, derived from *“Cyperus*,*”* referring to the genus of the plant *Cyperus alternifolius*, from which the species was recovered).

The novel species exhibited characteristics typical of the *Pseudocitrobacter* genus, including fermentative metabolism, non-hemolytic, facultative anaerobic growth, and a Gram-negative, short-rod cell morphology. It lacked spore formation and tested positive for catalase production but negative for oxidase activity. It demonstrated a remarkable ability to grow across a wide range of temperatures (4–45 °C), salinity levels (0–10% NaCl), and pH values (4–10). On TSA medium, it formed small, round, glistening colonies with a creamy white appearance. Acid is produced from glucose, D-cellobiose, D-maltose, D-mannitol, D-mannose, D-sorbitol, D-trehalose, and D-xylose, but not from adonitol, L-arabitol, saccharose, or raffinose. The strain tests positive for β-galactosidase, phosphatase, and tyrosine arylamidase, and negative for urease, β-glucosidase, and H₂S production. It reduces nitrate to nitrite but does not produce indole or acetoin. Lysine decarboxylase and ornithine decarboxylase are negative. Antimicrobial susceptibility testing revealed resistance to cefazolin and cefoxitin but sensitivity to other tested antibiotics. In contrast to all other *Pseudocitrobacter* species, the novel species exhibited negative citrate activity. Cyp38S^T^ showed distinct biochemical reactions from all identified *Pseusocitrobacter* species including the newly identified strain *P. limerickensis*, demonstrating uniquely positive D-sorbitol fermentation. Our strain could be also identified from *P. vendiensis* through acid production from malonate utilization, where *P. vendiensis* tested negative. Based on FAME GC-MS analysis, the major cellular fatty acids in Cyp38S^T^ are C_16:0_ (37.2%), C_17:1_ n-7 cis (21.8%), and C_18:1_ n-9 (10.3%), with additional amounts of C_14:0_ (6.8%), C_14:1_ (5.7%), and C_16:1_ (7.9%). The fatty acid profile is distinct from other *Pseudocitrobacter* species, with notable proportions of Myristoleic acid (C_14:1_), Palmitoleic acid (C_16:1_), cis-10-Heptadecenoic acid (C_17:1_ n-7 cis). The absence of C_12:0_ and C_19:0_ cyclo ω8c makes it more distinct from all other known *Pseudocitrobacter* sp. The draft genome sequence analysis showed a total size of 4,887,821 base pairs with a GC content of 53.5%. The novel species carries a unique CMY2/MIR/ACT/EC family class C beta-lactamase-coding gene and several efflux pump genes.

The type strain, Cyp38S^T^, was isolated from *Cyperus alternifolius* plant, Egypt and shows unique biochemical and fatty acid profiles compared to other species within the genus *Pseudocitrobacter*. The strain has been deposited in the Culture Collection Ain Shams University with the strain number CCASU-2024-73^T^.

## Electronic supplementary material

Below is the link to the electronic supplementary material.

Supplementary Material 1

## Data Availability

The Whole Genome Shotgun project has been deposited in the NCBI database under the BioProject number PRJNA1060458 and the genome assession number JAYMYY000000000.

## Abbreviations

TSA: Trypticase soy agar
WGS: Whole genome sequencing
COGs: Clusters of orthologous group
TYGS: Type strain genome server
GBDP: Genome BLAST distance phylogeny
OGRIs: Overall genome-relatedness indices
ANI: Average nucleotide identity
ANIm: Average nucleotide identity calculated by MUMmer
ANIb: Average nucleotide identity calculated by the alignment algorithms BLAST +
Tetra: Tetra-nucleotide signatures
dDDH: The digital DNA–DNA hybridization (dDDH)
BGCs: Biosynthetic gene clusters
KEGG: Kyoto encyclopedia of genes and genomes
KO: KEGG orthology groups
GC-MS: Gas Chromatography-Mass Spectrometry
FAME: Fatty acid methyl ester
